# Achieving High Resolution Timer Events in Virtualized Environment

**DOI:** 10.1371/journal.pone.0130887

**Published:** 2015-07-15

**Authors:** Blazej Adamczyk, Andrzej Chydzinski

**Affiliations:** Institute of Informatics, Silesian University of Technology, Gliwice, Poland; The University of Science and Technology of China, CHINA

## Abstract

Virtual Machine Monitors (VMM) have become popular in different application areas. Some applications may require to generate the timer events with high resolution and precision. This however may be challenging due to the complexity of VMMs. In this paper we focus on the timer functionality provided by five different VMMs—Xen, KVM, Qemu, VirtualBox and VMWare. Firstly, we evaluate resolutions and precisions of their timer events. Apparently, provided resolutions and precisions are far too low for some applications (e.g. networking applications with the quality of service). Then, using Xen virtualization we demonstrate the improved timer design that greatly enhances both the resolution and precision of achieved timer events.

## Introduction

Throughout the years time keeping techniques for common operating systems have evolved several times. Some changes were caused by the introduction of a new timer hardware into PC architecture. Processor and motherboard manufacturers have created several different devices to keep the track of time, in particular: the Programmable Interval Timer (*PIT*) [1], Real Time Clock (*RTC*) [2], Local Advanced Programmable Controller (*lapic*) [3], Advanced Configuration and Power Interface (*ACPI*) (also called *chipset timer*) [4], Time Stamp Counter (*TSC*) (see RDTSC instruction in [5]) and High Precision Event Timers (*HPET*) [6]. Each of the above was designed to serve a different purpose. Some provide advanced interrupt functionality while others are based on a simple counter register without any asynchronous notifications. Other changes in time handling techniques originated from architectural changes, like the introduction of Linux *Tickless* kernel mode. Previously, the kernel required a periodic timer interrupt to be delivered in order to work properly. The tickless mode allows the kernel to have longer inactivity periods and saves it from unnecessary interrupts and context switches while still maintaining good performance and time synchronization [7]. Another important modification to Linux timing functionality was the introduction of High Resolution Timers (*hrtimers*) [8]. This is a universal mechanism for scheduling timer events on currently available hardware thus allowing for a good resolution. It has become very popular and it is currently widely used among various kernel modules.

Today’s computer systems are often being virtualized to increase the hardware utilization and to decrease maintenance costs. Thus the programmers are faced with many new challenges related to the virtualization concept. Usually, in such environments one main operating system has a direct access to the hardware and is responsible for sharing it among other virtual machines. Naturally, this additional software layer influences the performance and may be crucial for the time-related functionality.

As for the related literature, the implementation of *hrtimers* in Linux operating system (without virtualization) is well described and discussed (e.g. in [8] or [9]). However, considering virtualized environments the emulation of timer hardware becomes a very important factor which influence is not well described. For instance, there is very little information available about the timer implementation in Kernel-based Virtual Machine (*KVM*). The authors of [10] while trying to achieve the virtualization with real-time processing explained and analyzed the timer mechanism but only briefly and with no measurements of the accuracy or precision. The VMWare VMM implementation of the timers is described in detail in [11]. Unfortunately, no measurements of the resolution or precision are presented either. In a VMWare virtual machine several different timer devices are being emulated. The emulation mechanism, time synchronization issues and the distribution of timer events among several virtual processors (VCPU) may have significant, as well as negative impact on the precision. Regarding Xen available literature is missing a detailed description of the Xen timer implementation. Moreover, we could not find any studies regarding Xen timer precision or accuracy. In [12] a detailed description of the Xen internals is presented, yet with only a brief explanation of the timer mechanism itself.

Depending on the application timer resolution and precision are not always considered as the most important. Several papers focus on decreasing timer resolution in virtualized environments in order to achieve other goals. In [13] for example the authors evaluate the influence of the timer events on the overall power consumption. In such a situation it is good to minimize the number of timer events and their processing complexity. Sometimes, the elimination of the fine grained timers in virtual environments can increase the security, as it was shown in [14]. In case of this study the situation is opposite—we are interested in high timer resolution and precision. As VMMs are general purpose systems, the potential applicability of precise timers is wide.

As it will be shown presented virtualization platforms provide timer events at a resolution of hundreds of microseconds. For some applications this resolution may be far too low. In networking, for instance, the transmission of a typical packet (1500 bytes) on a typical interface (1 Gb/s) takes only 12*μs* while the resolution of the timer events in the examined virtualization platforms may be a few orders of magnitude lower than that. Therefore, we cannot design applications with a high quality of service requirements, e.g. providing the total packet processing time below 20*μs*.

We faced this problem while implementing a time-based network scheduler for the virtualized networking infrastructure described in [15]. However, there are many other applications where high resolution timers may be required, e.g. CPU scheduling [16], automotive platforms [17] and others.

The purpose of this paper is twofold. Firstly, we present the measurements of the resolution and precision of the timer events in some commonly used virtual machine monitors: VMWare [18], VirtualBox [19], Qemu [20], Xen [21], and KVM [22]. Secondly, we propose our own implementation of the virtual timer interrupts for Xen which provides far better resolution and precision comparing to the original measurements of all five platforms.

The detailed outline of the paper is as follows. In Section *Evaluated Virtualization Systems*, all five evaluated virtualization platforms are briefly described. Section *Experiments* presents the performed measurements of timer event resolution and precision for all mentioned VMMs. We also compare and discuss the results in this section. Beginning with Section *Xen VMM* we focus on Xen VMM by describing its architecture and the timer implementation. Then, in section *New High Resolution Timer*, we present how Xen sources can be modified to achieve much better resolution and precision by creating a separate timer mechanism. Naturally, measurements of the resolution and precision of the proposed implementation are also presented. Finally, the conclusion is presented in section *Conclusion*.

### Virtual Machine Monitors

Virtualization systems fall into two categories: full virtualization and paravirtualization. Before hardware virtualization support technologies (namely the Intel VT-x and AMD SVM, later called AMD-V), the x86 architecture was not easy to virtualize (the architecture did not meet the virtualization requirements stated by Popek and Goldberg in [23]), as some crucial CPU instructions could be executed from outside the privileged mode without causing a trap. To tackle this problem some virtualization engines (e.g. VMWare) use the so called “binary rewrite” method (see [12]) which results in significant performance penalty, due to all forbidden instructions have to be detected and rewritten at run-time. On the other hand, paravirtualization (e.g. Xen) approaches the problem differently by rewriting the problematic instructions at the design time—it means that the guest operating system has to be modified in advance to work properly. Xen was one of the first virtualization platforms that used the concept of paravirtualization.

Both approaches have their advantages and disadvantages. Full virtualization is much more portable and allows to virtualize any operating system, but at the expense of performance. Paravirtualization does not require the emulation of the hardware and allows many hardware-related operations to be performed more efficiently.

Nowadays, after the virtualization support technologies were introduced (thus eliminating the mentioned x86 virtualization problems) the distinction between full and paravirtualization has significantly blurred. For example, Xen utilizes Intel VT-x and AMD-V to support full virtualization. Kernel-based Virtual Machine (KVM), which was designed to implement strictly full virtualization, has started to use paravirtual drivers to achieve better IO operations performance.

Another factor which differentiates Virtual Machine Monitors is their architecture. Some VMMs (like Xen, VMWare vSphare Hypervisor—previously called VMWare ESXi, and Microsoft Hyper-V) use a dedicated small operating system called hypervisor that is responsible for the virtualization of the main resources, such as CPU and memory. Other VMMs (such as KVM, Qemu, VirtualBox, VMWare Workstation/Player or Microsoft Virtual PC) utilize an existing host operating system to perform this task.

Usually, in hypervisor-based virtualization the user interacts first with the virtual machine, not the hypervisor itself, as it does not provide any user interface. Such small purpose-built hypervisors allow for better implementation without any limits placed by an existing generally purposed operating system. On the other hand, all hardware related mechanisms need to be reimplemented which may cause problems with stability and efficiency. In contrast, the VMMs based on an existing operating system inherit the implementation and corrections from the operating system which is usually mature and well tested.

## Evaluated Virtualization Systems

For our experiments we chose five free virtualization platforms which represent mentioned groups: Xen—a hypervisor based paravirtualization, KVM—Linux kernel-based full virtualization with the use of the hardware virtualization technology, Qemu—Linux quick emulator with full software virtualization, VirtualBox—Open Source full virtualization software maintained by Oracle company, and VMWare Player—host operating system based full virtualization technology. The evaluated products are widely used around the world and certainly are among the most popular virtualization platforms. A short description of each will be presented in the following sections.

### VMWare Player

VMWare Player is a free version of the VMWare virtualization platform which is based on the same core functionality as VMWare Workstation. It is available for Windows and Linux based host operating systems. According to the End User License Agreement [24] it can be used for personal and non-commercial purposes, or for distribution or other use by written agreement. The first version of VMWare Player was released in June 2008. It was based on version 6 of VMWare Workstation. The roots of VMWare Workstation date back to 1999, when the initial version was released. The latest stable release of VMWare Player at the time of writing this article is version 7.1.0.

VMWare software can be run on an existing host operating system. It is possible to run VMWare virtual machines on Microsoft Windows, Linux and Mac OS X host operating systems. Because it performs a full virtualization, by means of emulation and binary rewriting, it is possible to run any x86 and x86–64/AMD64 compatible operating systems [25].

### VirtualBox

VirtualBox is an open source full virtualization platform. In terms of architecture and design it is very similar to VMWare products. The first version of VirtualBox was released by Innotek GmbH in 2007. Since the beginning an Open Source Edition of VirtualBox has been available with the GNU General Public License, version 2. The project was bought by Sun Microsystems in 2008 and later in 2010 with the acquisition of Sun by Oracle it was renamed to “Oracle VM VirtualBox”.

VirtualBox supports the common host operating systems: Microsoft Windows, Mac OS X, Linux and Solaris. Similarly to VMWare, the full virtualization is achieved by binary rewriting ring 0 privileged instructions using the Code Scanning and Analysis Manager (CSAM) and the Patch Manager (PATM). The latest stable release of VirtualBox is 4.3.26.

### Qemu

The Quick Emulator called Qemu is an open source project which allows for emulation of different hardware platforms and run fully virtualized guest operating systems. It can emulate CPU of the following platforms: x86, MIPS, 32-bit ARMv7, ARMv8, PowerPC, SPARC, ETRAX CRIS and MicroBlaze. It can also emulate hardware devices to let for full support of common operating systems.

Qemu by itself can be used to run userspace programs compiled for different platforms or to fully emulate a computer system with all necessary hardware. Qemu is also employed in Xen and KVM in hardware (other than CPU) emulation tasks. The Qemu project was started by Fabrice Bellard in 2004, is available for free, and is mainly licensed under GNU General Public License. It can be compiled and run on most major host operating systems. Current stable version of Qemu is 2.2.0.

### KVM

KVM (Kernel-based Virtual Machine) is a virtualization platform based on the Linux kernel in the role of a hypervisor. KVM is an open source project established in 2006 by Qumranet, Inc. and currently maintained by the Open Virtualization Alliance. It is licensed with either GNU General Public License or GNU Lesser General Public License. It provides full virtualization capabilities but only for processors with hardware virtualization support (Intel VT-x or AMD-V). Although KVM provides only full virtualization of CPU and memory, it additionally allows the use of different kinds of IO device virtualization:

- Emulation—the devices are fully emulated which allows for great portability, but at the expense of the performance.
- Paravirtualization—the device drivers used in the virtual machine are aware of being virtualized and utilize the virtualization interface to perform IO operations much more effectively; this approach requires the use of special drivers for the guest operating system.
- Pass-through—the device is assigned to a certain virtual machine and is not intended to be shared.

KVM is a kernel module and is a part of Linux kernel sources since version 2.6.20. The module exposes the */dev/kvm* interface which can be used by user-space programs (e.g. *qemu-kvm*) to perform virtualization.

### Xen

The initial release of Xen hypervisor was created at Cambridge University in 2003. The product was initially supported by XenSource Inc. which in 2007 was acquired by Citrix Systems, a company that supports the development of a free version of Xen but additionally sells an enterprise edition to commercial clients. Currently, Xen open source software called Xen Project is maintained as a collaborative project by the Linux Foundation consortium.

Xen provides a hypervisor-based virtualization. Initially, it allowed for paravirtualization only but with the introduction of hardware virtualization extensions, Xen also allows full virtualization mode. The core of Xen software is the hypervisor. It is a purpose-built, small operating system that provides virtualization of CPU and memory and delegates control to the first, privileged virtual machine called *Domain0*. The user may run and manage all virtual machines from within *Domain0*. All IO devices are handled by privileged domains which use paravirtual drivers to share them with other virtual machines. Such architecture makes the system very stable, as the hypervisor does not handle device drivers or other functionalities which usually cause problems.

## Experiments

To check the resolution and precision of the clock event devices we performed several tests. In all of them we used Linux Gentoo 3.17.8 as both host and virtual machine operating system. We performed the experiment on several hardware platforms but the results presented here are based on an Intel Xeon E5645 @ 2.4GHz, 24GB RAM with Supermicro X8DTN motherboard. The timer events were scheduled using *hrtimers* within a virtual machine. We have created a special Linux kernel module which was used to perform and gather the results of measurements.

In the experiments we installed version 6.0.0 of the VMWare Player, version 4.3.18 of the VirtualBox and version 2.1.2 of the Qemu, all installed from Linux Gentoo repository (we used the most recent stable versions of all three products available in the repository). For KVM, we used the version of KVM available with the host kernel version i.e. 3.17.8. Finally, to test Xen we used version 4.5.0 of Xen hypervisor. Additionally, we have also performed the experiment on a plain Linux Gentoo 3.17.8 without using virtualization.

Considering Xen, it is important to note that the default Xen implementation sets a slop variable (*timer_slop*) that allows merging of the timer events from certain time ranges into one interrupt. The default value of *timer_slop* is set to 50*μs*, what is good for general purposes and unloads the processor from unnecessary interrupt handling and context switching. Moreover, the Xen sources of Gentoo kernel version 3.17.8 also use a similar slop constant (*TIMER_SLOP*) which is set to 100*μs*. Both slop settings can be changed to obtain more fine-grained timer events with the cost of more interrupts and context switches. For the purpose of this experiment, we have changed the slop value to 1*ns* in the hypervisor and in the guest domain kernel sources. Without changing this value, the timer events in Xen virtual machines were merged and fired with the resolution of 100*μs*.

The presented measurements consist of three parts which are supposed to show what is the timer resolution and precision under the different circumstances. In the first part, all virtualization platforms were tested in a scenario of an idle system—the system was not performing any intense IO or CPU operations. In the second test, the system was loaded with a set of CPU loads. Finally, the last part presents the influence of intense IO disk operations which were causing many additional interrupts to be handled by the processor at the same time.

The results show that heavy CPU load does not influence the precision of the timer events very much. This was to be expected as the timer events are handled as interrupts. Thus, the incoming interrupts result with CPU preemption no matter what is the actual load level of the CPU. The case is different when the system is loaded with other interrupts.

Historically Linux kernel was dividing the interrupt handling routines into ‘slow’ and ‘fast’ handlers. This allowed ‘slow’ interrupts to be preempted by ‘fast’ ones in order to improve the performance of time critical functions. Such distinction between the interrupts originated from not properly designed device drivers that were consuming too much CPU time inside the interrupt handlers. This was systematically fixed by removing the time consuming parts of the drivers into separate kernel tasklets running outside of the interrupt handlers. Finally, since version 2.6.35 the Linux kernel switched all interrupt handlers to be run as ‘fast’ (the *IRQF_DISABLED* flag was marked as deprecated) meaning that an interrupt cannot be preempted. In general this was a very good decision as it simplified the interrupt handling mechanisms and freed the kernel from handling nested interrupts. Considering high resolution timers however, this change could have a negative influence on timer precision. Obviously, even the fast interrupt handlers still consume some CPU time. Thus, some other interrupts occurring right before the timer event on the same CPU may introduce additional delays.

The third part of the experiments proves that other interrupts may have a strong impact on the timer precision. We chose to create IO interrupts by reading the disk, but the problem could have been demonstrated using other type of interrupts as well.

It is worth noting the following consequence of the fact that the timer performance depends on the interrupts: the number of virtual machines running on the hypervisor does not influence directly the timer resolution and accuracy. They are influenced only by the number of and types of interrupts generated by them.

The detailed results are presented in the following sections.

### Idle system

Firstly, in an idle system, 4500 timer events were scheduled every *d* microseconds. During our study we have performed many different experiments with different values of *d*. In this article we present only the border values of *d* which were hard to achieve by most of the tested platforms.

For every scheduled event the actual time interval was measured. Then the mean value, the sample uncorrected standard deviation, and the 99% confidence intervals were computed.

**Fig 1:**
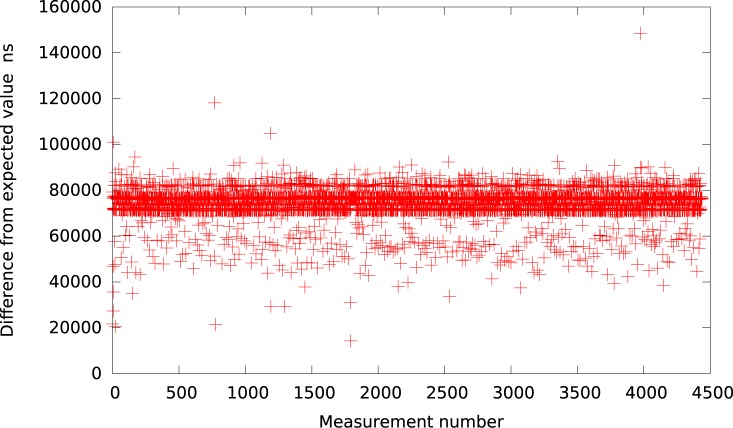
The difference between the measured timer interval and its expected value 50*μs* in VMWare (idle system).

All results are gathered together in Table 1 on page 17. A detailed representation for VMWare Player for an interval duration of 50*μs* is additionally depicted in Fig 1. The graph for VMWare is similar for all measurements greater than 50*μs*, with the measured time being usually delayed by around 80*μs* from the expected value. Such results could be expected because the timer device is being emulated by VMWare software which causes the additional delay.

**Table 1:** Summary of all measured statistics for each timer version discussed.

Experiment	Timer implementation	Mean measured time [*μs*]	Standard deviation [*μs*]	Confidence interval for the mean *α* = 99%
**timer setting = 150*μs* idle system**	VMWare	228.999	7.987	(228.764,229.234)
	VirtualBox	218.370	71.147	(216.268,220.473)
	Qemu	190.415	4.492	(190.282,190.548)
	KVM	164.593	1.029	(164.562,164.623)
	native Xen	150.213	0.282	(150.204,150.221)
	new timers	150.151	0.175	(150.146,150.156)
	Linux kernel	150.025	0.073	(150.023,150.027)
**timer setting = 100*μs* idle system**	VMWare	175.908	9.867	(175.617,176.198)
	VirtualBox	159.232	9.252	(158.959,159.506)
	Qemu	140.259	3.832	(140.145,140.372)
	KVM	114.087	0.753	(114.064,114.109)
	native Xen	100.221	0.339	(100.211,100.231)
	new timers	100.074	0.118	(100.071,100.078)
	Linux kernel	100.103	0.070	(100.101,100.105)
**timer setting = 50*μs* idle system**	VMWare	123.951	7.110	(123.742,124.161)
	VirtualBox	97.749	7.633	(97.524,97.975)
	Qemu	82.768	9.912	(82.475,83.061)
	KVM	65.310	1.526	(65.265,65.355)
	native Xen	50.108	0.085	(50.105,50.110)
	new timers	50.071	0.094	(50.068,50.073)
	Linux kernel	50.056	0.083	(50.053,50.058)
**timer setting = 10*μs* idle system**	VMWare	24.497	4.269	(24.371,24.622)
	VirtualBox	54.370	46.192	(53.005,55.735)
	Qemu	66.450	11.418	(66.113,66.788)
	KVM	21.762	2.637	(21.685,21.840)
	native Xen	10.127	0.078	(10.125,10.130)
	new timers	10.060	0.062	(10.058,10.062)
	Linux kernel	10.076	0.175	(10.070,10.081)
**timer setting = 150*μs* system with heavy IO**	VMWare	669.971	10.026K	(374.986,964.957)
	VirtualBox	214.700	63.933	(212.811,216.589)
	Qemu	232.981	84.149	(230.494,235.467)
	KVM	177.355	21.907	(176.708,178.003)
	native Xen	152.796	10.177	(152.496,153.097)
	new timers	150.172	0.182	(150.167,150.178)
	Linux kernel	152.091	7.513	(151.869,152.313)
**timer setting = 100*μs* system with heavy IO**	VMWare	485.224	4.266K	(359.720,610.727)
	VirtualBox	156.717	13.723	(156.311,157.122)
	Qemu	177.905	80.585	(175.524,180.287)
	KVM	119.712	9.869	(119.421,120.004)
	native Xen	105.312	17.797	(104.786,105.838)
	new timers	100.151	0.168	(100.146,100.156)
	Linux kernel	101.454	6.340	(101.267,101.641)
**timer setting = 50*μs* system with heavy IO**	VMWare	427.037	3.558K	(322.353,531.722)
	VirtualBox	99.504	14.097	(99.088,99.921)
	Qemu	116.652	67.896	(114.646,118.659)
	KVM	76.398	21.001	(75.778,77.019)
	native Xen	54.214	17.628	(53.694,54.735)
	new timers	50.054	0.156	(50.049,50.058)
	Linux kernel	51.767	8.180	(51.525,52.009)
**timer setting = 10*μs* system with heavy IO**	VMWare	491.723	3.960K	(375.207,608.239)
	VirtualBox	58.146	45.545	(56.800,59.492)
	Qemu	71.087	32.459	(70.128,72.046)
	KVM	25.015	12.234	(24.653,25.376)
	native Xen	11.274	10.643	(10.959,11.588)
	new timers	10.053	0.096	(10.050,10.056)
	Linux kernel	10.568	5.071	(10.418,10.718)

**Fig 2:**
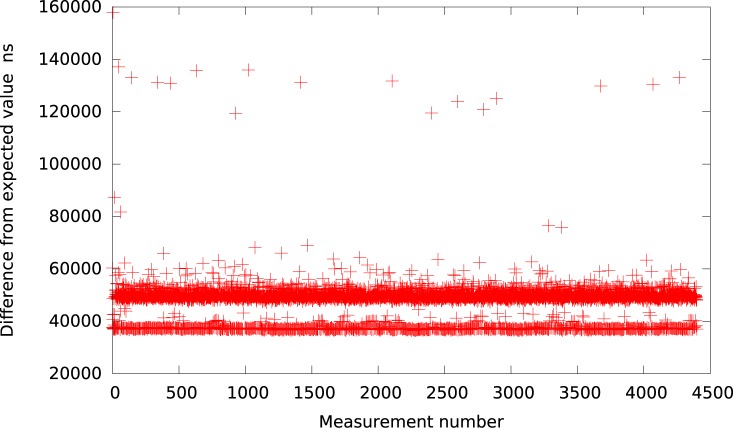
The difference between the measured timer interval and its expected value 50*μs* in VirtualBox (idle system).

Analogically, the graph for VirtualBox is presented in Fig 2. As it can be observed by looking at Table 1, VirtualBox is usually (for all delays greater than 10*μs*) delayed by about 37 − 60*μs* from the expected value what is slightly better in terms of resolution than VMWare Player but still very inaccurate and imprecise.

**Fig 3:**
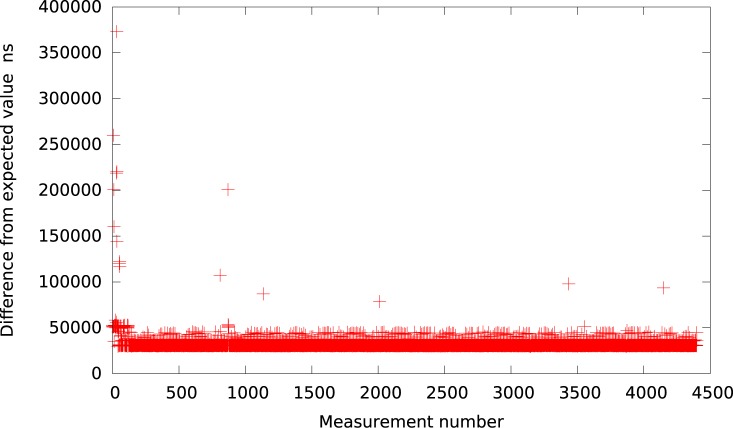
The difference between the measured timer interval and its expected value 50*μs* in Qemu (idle system).

Further, the Qemu provides again a slightly better resolution. The results, presented in Fig 3, show that for the intervals greater than 50*μs* the timer events are usually delayed by about 40*μs*. Thus, we may summarize that all emulated userspace full virtualization platforms (i.e. VMWare Player, VirtualBox and Qemu) provide similar timer resolution at the level of tens of microseconds within an idle system.

**Fig 4:**
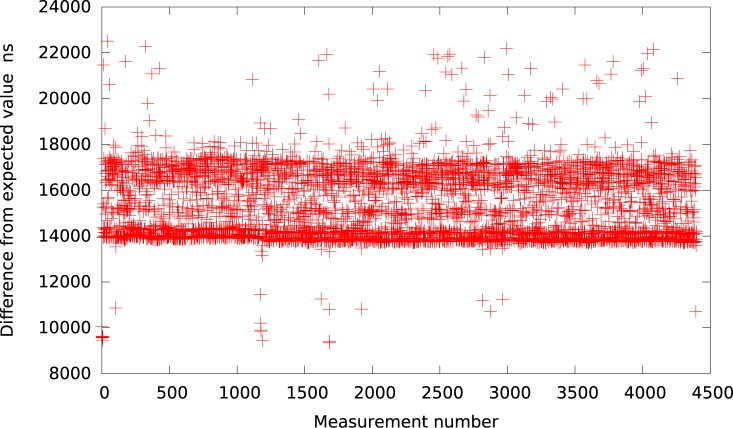
The difference between the measured timer interval and its expected value 50*μs* in KVM (idle system).

Continuing, the graph for KVM for an interval of 50*μs* is shown in Fig 4. It can be easily observed that the resolution of the timer events in KVM is more fine-grained than in the case of VMWare Player, VirtualBox and Qemu. The difference between the expected and measured timer intervals is at the level of 12 − 16*μs*.

**Fig 5:**
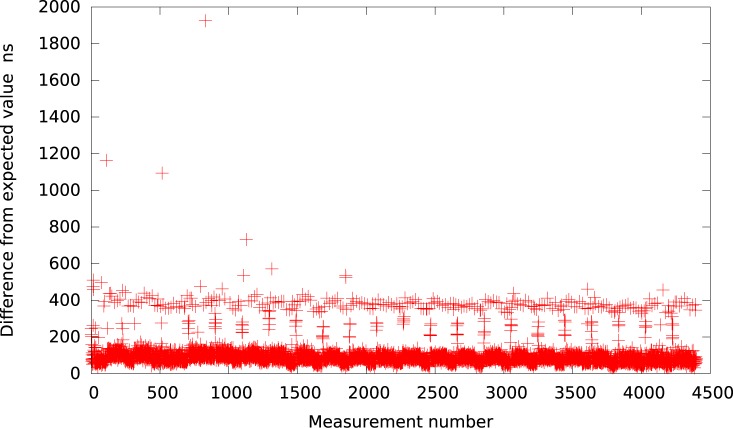
The difference between the measured timer interval and its expected value 50*μs* in Xen (idle system).

Next we performed the same experiment in a Xen based virtual machine. Again the results are presented in Table 1 and for an interval duration of 50*μs* additionally depicted in Fig 5. As one can observe, the resolution of Xen is much better when compared to KVM and all other tested virtualization platforms.

**Fig 6:**
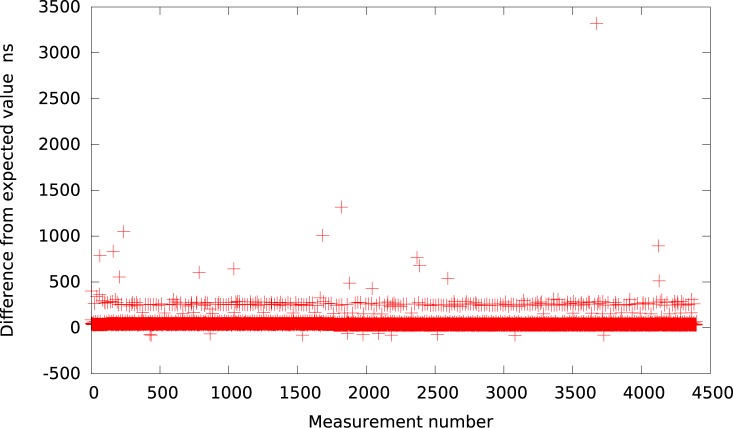
The difference between the measured timer interval and its expected value 50*μs* in Linux kernel without virtualization (idle system).

Finally, the experiment was also repeated within bare Linux kernel without any virtualization. The results are depicted in Fig 6. As it was to be expected the resolution is at a similar level but the precision is even better than in case of Xen. The standard deviation for a bare Linux kernel is at the level of one-tenth of a microsecond.

### Influence of CPU load

Results presented in the previous section are valid for an idle system—i.e. a system without any substantial IO operations or CPU load. As it was already stated intense CPU load should not have a significant influence on the timer resolution because the timer events are being executed as interrupts and thus the CPU is preempted independently of the current CPU state. Nevertheless, we decided to perform an additional experiment to show that this is in fact the case.

The test for 10*μs* similar to the one described in the previous section was repeated for Xen platform for 3 different values of CPU load. The CPU load was generated on all 24 cores of the machine with the use of Linux *stress-ng* tool [26]. The results are presented in Table 2.

**Table 2:** CPU load influence.

CPU load	Timer implementation	Mean measured time [*μs*]	Standard deviation [*μs*]
**30% CPU load**	native Xen	10.217	0.375
	new timers	10.039	0.067
**60% CPU load**	native Xen	10.188	0.398
	new timers	10.148	0.262
**100% CPU load**	native Xen	10.151	0.313
	new timers	10.100	0.312

As it can be observed the resolution is similar to the one obtained in an idle system and stays at the level of one microsecond. The additional load has some small influence on the precision of the timer events but the effect is marginal. It can be related with several aspects. Firstly, when a processor is under load the interrupt may occur more frequently under different CPU states: idle or running. In both cases the number of instructions to be executed before the actual timer handling routine may vary. Thus, the standard deviation is bigger and the timer duration is less precise. Secondly, additional variation may be introduced because of the power-saving functions, as well as temperature control logic.

### Influence of other interrupts

**Fig 7:**
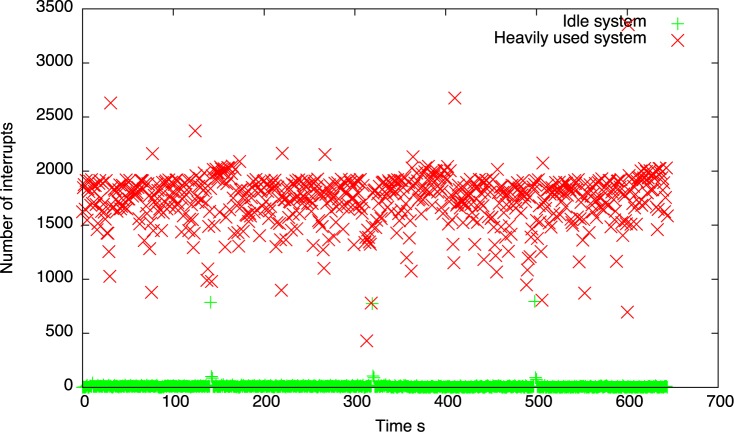
The number of interrupts per second in the idle system and in the system with heavy IO load.

Similarly to the idle system test experiments with four values of *d* and 4500 timer events were repeated in a system with heavy IO usage. Namely, heavy disk operations within the virtual machine were triggered in attempt to induce a negative effect of the other IO interrupts on the timer accuracy and precision. This was achieved by clearing the disk caches and using the *dd* Linux utility to read data from disk. To make the difference more visible the disk driver interrupt was pinned to the same processor as the timer event device using the Linux *smp_affinity* interrupt mask. To present the impact of disk read operations on the number of interrupts of a single CPU, we gathered the interrupt statistics for both, the idle system and the system with heavy IO usage. Fig 7 presents the number of interrupts per second in both cases.

The average number of the interrupts per second for an idle system is 11.52 and for the system with heavy IO load is 1733.35. As we can see, due to the disk read operations many additional disk interrupts are handled by the CPU. According to the arguments presented in Section *Experiments*, if these interrupts are executed on the same CPU just before the scheduled timer interrupt, then they cause the timer events to be delayed.

The results of the experiment for the system with heavy IO load are again presented in Table 1 on page 17. As it can be observed the additional interrupts have significant impact on the resolution and precision of the timers in all virtualization platforms.

**Fig 8:**
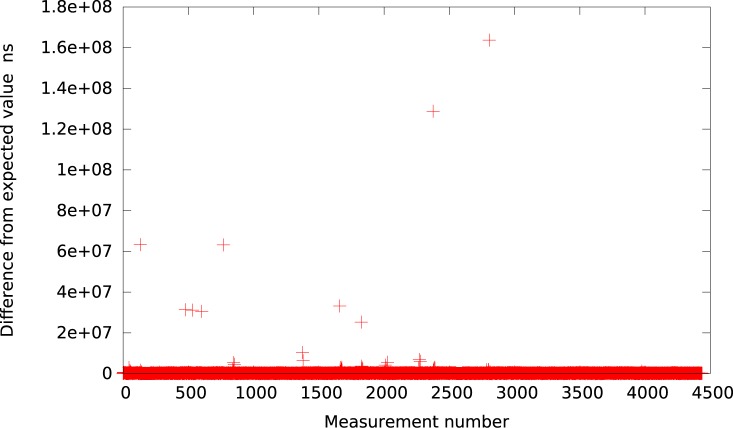
The difference between the measured interval and its expected value 50*μs* in VMWare (system with heavy IO load).

For VMWare, the influence of IO operations is very noticeable. First of all, the resolution seems to be out of the microsecond scale (it is around half of a millisecond). The precision is also very low. Detailed results presented in Fig 8 additionally show that there were few outlier events triggered after tens to hundreds of milliseconds, which may be unacceptable for some time depended applications.

**Fig 9:**
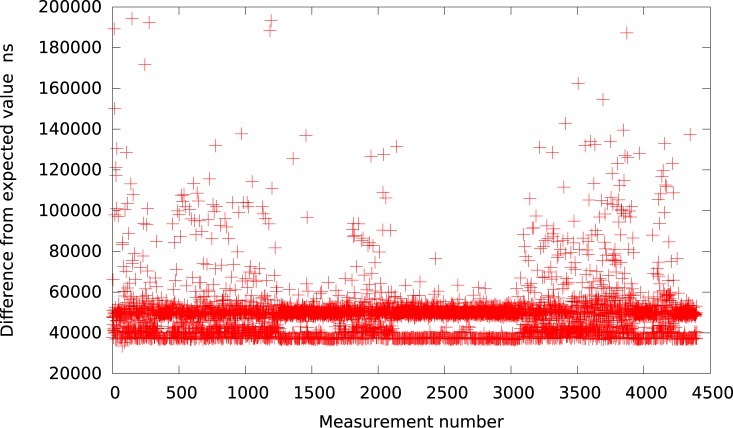
The difference between the measured interval and its expected value 50*μs* in VirtualBox (system with heavy IO load).

As far as VirtualBox is concerned the influence of additional IO interrupts is also visible. Again, the detailed results for 50*μs* interval are presented in Fig 9. In contrast to VMWare there are no outliers above 200*μs*. This makes the standard deviation much smaller. There are however more values between 60 − 200*μs* comparing to the idle system which are probably caused by the additional interrupts.

**Fig 10:**
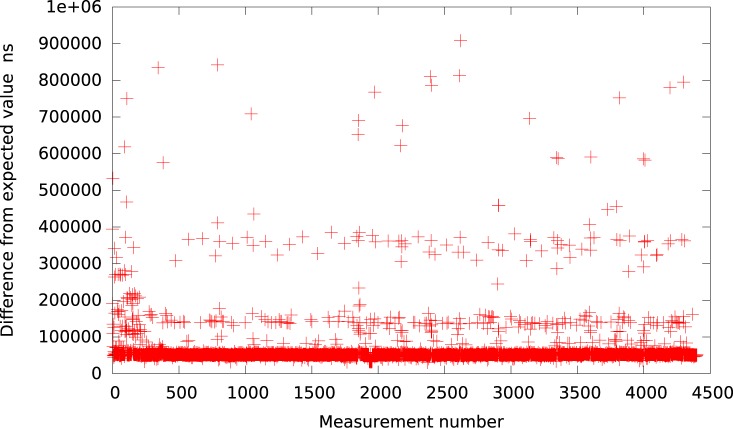
The difference between the measured interval and its expected value 50*μs* in Qemu (system with heavy IO load).

Qemu results are presented in Fig 10 as well as in the Table 1. Here, similarly to VMWare the influence of IO interrupts is clearly visible. The impact is not as strong as in case of VMWare but much stronger than in VirtualBox. The standard deviation is from around 3 (in the best case) to around 21 (in the worst case) times greater comparing to the idle system.

**Fig 11:**
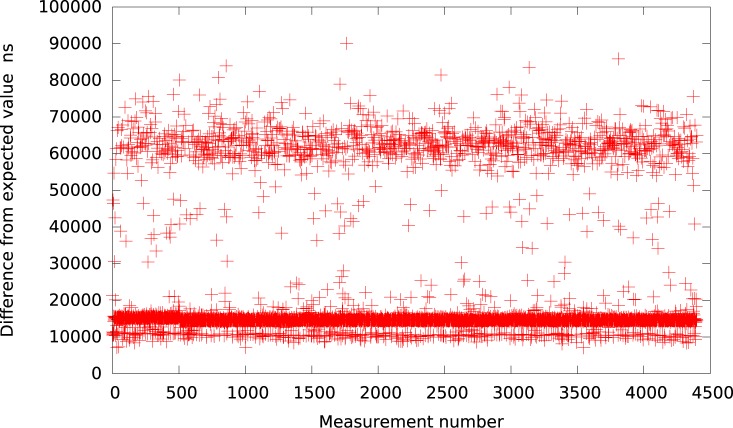
The difference between the measured interval and its expected value 50*μs* in KVM (system with heavy IO load).

In the case of KVM the results for a system with high IO load presented in Table 1 and in Fig 11 show that the majority of events occur at around 16*μs* (similarly to the idle system case) but there are also many values concentrated at the level of 55 − 70*μs* what is probably caused by the additional disk interrupts. To sum up, looking at the standard deviation we may conclude that the KVM timer events precision is from around 5 (in case of 10*μs* intervals) to around 21 (for 150*μs* intervals) times worse than in case of an idle system.

**Fig 12:**
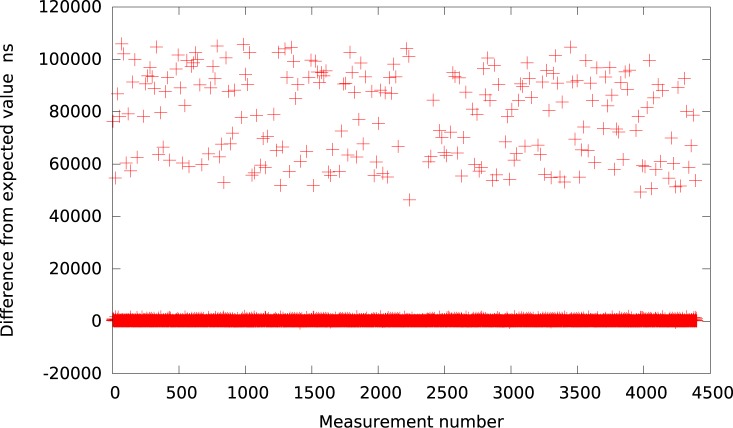
The difference between the measured interval and its expected value 50*μs* in Xen (system with heavy IO load).

The heavy IO operations also have an influence on the Xen timer events. As it is presented in Table 1 and Fig 12 the resolution is maintained but the precision is worse than in case of the idle system. There are multiple events over 50*μs*, which may be unacceptable for some applications. Nevertheless, Xen still proves to be better in resolution and precision when compared to all other virtualization platforms.

**Fig 13:**
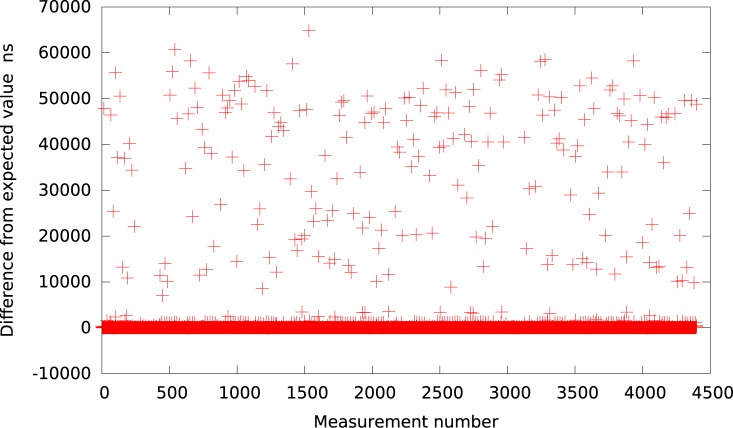
The difference between the measured interval and its expected value 50*μs* in Linux kernel without virtualization (system with heavy IO load).

Linux kernel without virtualization shows similar behavior to Xen. The results are also presented in Table 1 and Fig 13. Comparing to Xen the bare Linux kernel provides around 2 times better precision of the timer events in the case of a system with heavy IO load.

As we can see, heavy disk operations cause additional negative effects on the timer precision in all of the tested virtualization platforms. The effect is observed most significantly in VMWare and Qemu. The least affected were Linux kernel without virtualization and Xen.

Taking these results into consideration, we can say that among all the examined virtualization environments the most accurate timer events can be obtained by using Xen virtualization. However, modification of the *timer_slop* variables is required. Unfortunately, this change affects the number of delivered timer interrupts in all *hrtimer* usages inside all the virtual machines. Without this modification, Xen timer events have resolution of 100*μs*.

Summarizing, we can state that achieving the timer events with resolution of a about ten microseconds with high precision in the heavy IO load is not possible in any of the examined virtualization platforms. Even without the virtualization, the Linux kernel does not provide good precision with high IO loads. To solve this problem we decided to create a separate timer implementation in Xen which would be used only for special purposes, providing better resolution and precision while not affecting the overall system performance, and other hrtimer events.

In the following sections, we will describe the general Xen architecture and the timer event device implementation. Further we will show how we have implemented our own timer event device and present an evaluation of this implementation similar to the experiments shown above.

## Xen VMM

Xen was created to virtualize x86 architecture with good performance thanks to paravirtualization. As it has been already stated, this approach has several advantages over full virtualization mode. First of all, there is no run-time binary rewriting. Additionally, as the guest operating system requires to be modified, the changes can also eliminate certain device emulation, improving the performance even further.

**Fig 14:**
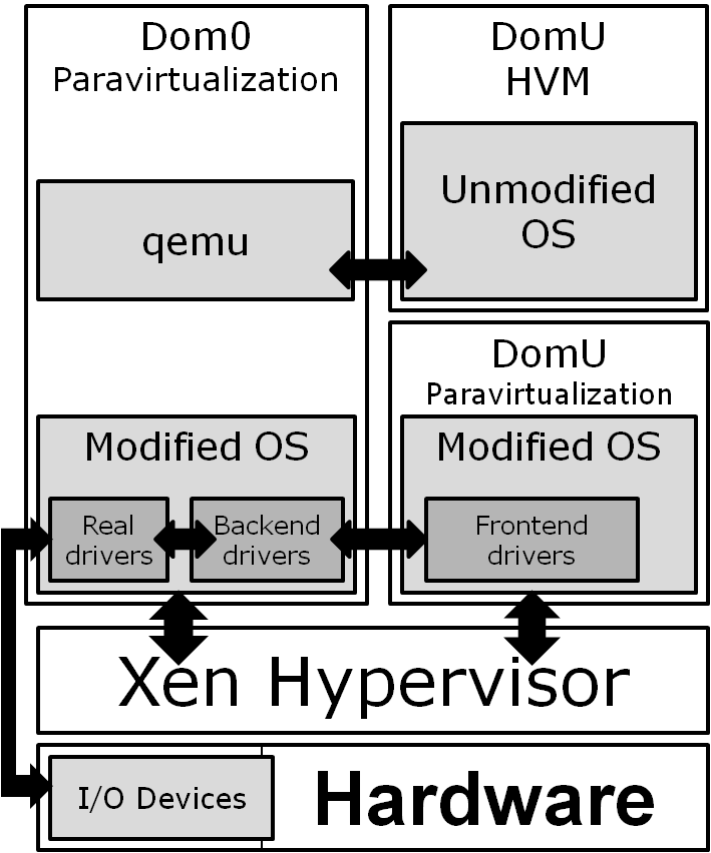
Xen VMM architecture.

Additionally Xen was one of the first virtualization platforms with hypervisor-based architecture. Instead of running an existing operating system directly on the hardware (i.e. in *Ring0*), it uses a small *hypervisor*, see Fig 14. After start, the hypervisor runs the first virtual machine, *Domain0*, in a less privileged mode (depending on architecture this could be *Ring1* or *Ring3*). In fact, this is the first OS that the user has contact with, because the hypervisor does not contain any user interfaces. The *Domain0* is a privileged virtual machine (VM) which can control and manage the hypervisor, and has a permission to access the IO devices. As *Domain0* can run any of the supported operating systems, it can use the existing device drivers to access the real devices. Moreover, it is responsible for sharing them with other VMs. This is achieved by the *Backend/Frontend* drivers (see Fig 14). Such design provides good reliability and stability of the system. The hypervisor controls only the CPU and memory, while the device drivers (which are the cause of most kernel errors) are separated from the hypervisor. Therefore their errors do not influence other VMs stability. Xen can be configured also to provide device access to certain VMs called *Driver Domains*. In this way the administrator can separate even a single device driver, so that a fatal system error in one of the driver domains would not affect any other.

### Xen timer implementation

In order to run a virtual machine, the VMM has to provide timer events to the guest operating systems. In contrast to other VMMs that achieve this through emulation of the existing timer devices, Xen again uses paravirtualization. The modified guest kernel contains a new *Clock Event Device* which schedules a timer interrupt in the hypervisor using *hypercalls* (see [12] or [27]). Further, the hypervisor sets local APIC timer accordingly. Of course, such an implementation requires not only a very fast delivery of the interrupt into the VM, but also some control mechanism for the scheduled events from all virtual machines.

#### Timers in the hypervisor

The Xen hypervisor uses local APIC for scheduling the timer events. The virtualized event device can work in two modes: *singleshot* and *periodic*. In order to schedule a new event, the VM has to call the *VCPUOP_set_singleshot_timer* or *VCPUOP_set_periodic_timer* hypercall accordingly. All scheduled timer events are stored in two data structures: (faster) heap and (slower) linked list. Each time the *lapic* interrupt occurs, the handler executes the expired timer events and, at the end, schedules next *lapic* interrupt looking for the earliest deadline timer. To improve the performance and lower the number of the timer interrupts, a *timer_slop* parameter is used. It denotes the amount of the time (in nanoseconds) that the timer can be late. All timers from such interval will be executed in a single *lapic* interrupt. The *timer_slop* is set by default to 50000*ns*.

#### Virtual timer interrupt

All interrupts in Xen are handled by the hypervisor. It then delivers them to guest OSs using so called *event channels* [12]. There are three types of the events: Physical IRQ, Virtual IRQ (VIRQ) and inter-domain events. Different hardware interrupts are delivered using Physical IRQs event channels. This is done to make the standard device drivers work correctly and map real IRQs inside the guest kernel. In the case of virtual devices, such as the aforementioned timer event device, the Virtual IRQs are used. There is a special virtual timer interrupt called *VIRQ_timer*.

**Fig 15:**
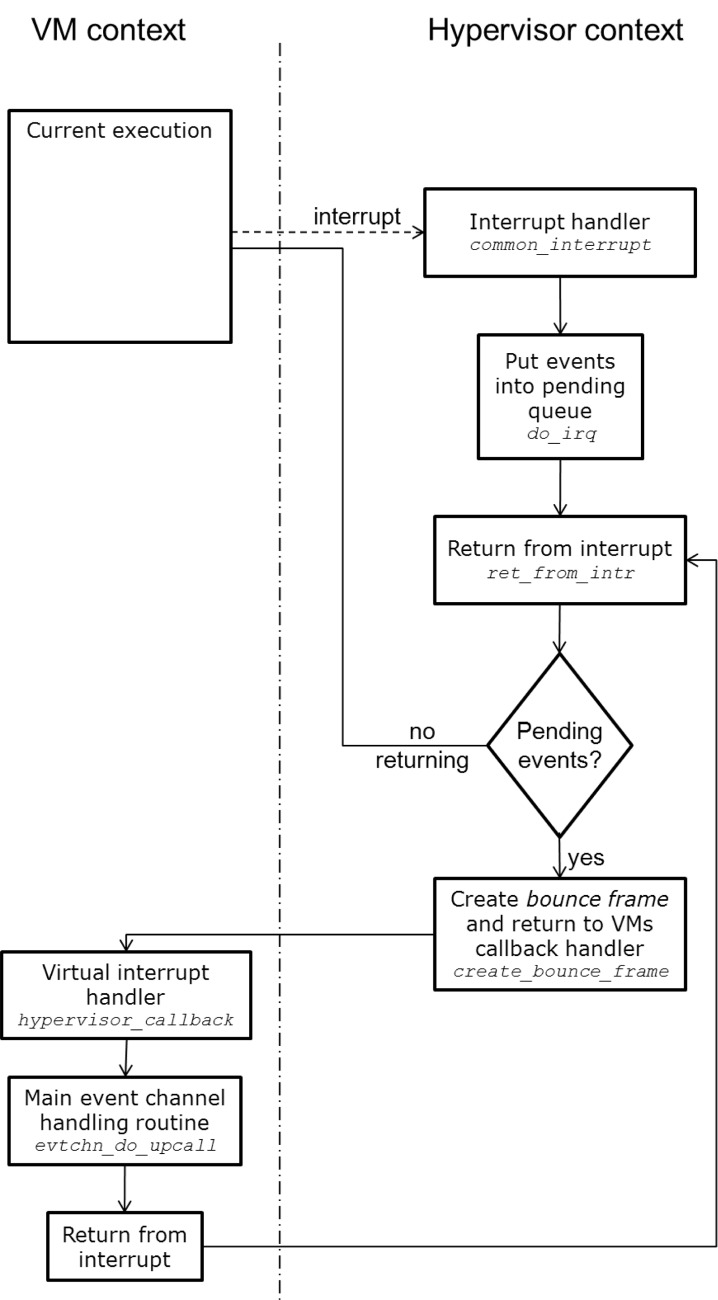
Simplified hardware interrupt handling process with propagation to VM using event channel.

A simplified process of handling hardware interrupt and delivering it to the VM using an event channel is presented in Fig 15.

Inside the hypervisor, all hardware interrupts are handled using the *common_interrupt* routine (defined in *xen/arch/x86/x86_64/entry.S* file). This macro is a wrapper for the real handler. It performs common interrupt tasks (like saving registers before executing the handler) but also returns to the VM context afterwards. The *ret_from_intr* assembly routine is used to execute the pending event channel requests and, at the end, return to the previously executed VCPU process. This is actually done every time the hypervisor returns to certain VCPU context, no matter what the cause of previous preemption was (it could be the mentioned hardware interrupt, but also Xen CPU scheduler preemption for different VCPU), to allow the VCPU receive the pending events as soon as possible. If an event is pending for the returning VCPU then the hypervisor has to return to the event handling routing in guest VM. This is done by creating a special *bounce frame* on the guest stack, so that the return will restore the instruction pointer to the event handling function (usually called *xen_hypervisor_callback*—the guest OS, while initiating has to register it using the *CALLBACKOP_register* operation of the *HYPERVISOR_callback_op* hypercall) with appropriate arguments. Further, the context switches to guest OS and executes the xen_hypervisor_callback routine which is a wrapper around the *xen_evtchn_do_upcall* function. The latter, using the arguments received in the bounce frame from the hypervisor, executes an appropriate handler for the given event channel.

As it was already stated, the timers in Xen are paravirtualized using a new clock event device (usually called *xen_clockevent* defined in *xen/time.c*). This virtual clock event device registers an event channel handler for the VIRQ_timer virtual interrupt. Further it becomes the default clock event device and thus it is used by all event related kernel mechanisms (one of them are *hrtimers*). Every time the timer event is scheduled the device sends the *VCPUOP_set_singleshot_timer* hypercall setting the next *VIRQ_timer* interrupt time.

Such a design has some drawbacks which may affect the timers resolution and precision. Firstly, all the scheduled timers are delivered using the same mechanism and the same virtual interrupt. In practice we need much less fine grained timers for some purposes than for the other, so using the same high resolution mechanism may result in decreased accuracy for the crucial applications. Additionally, other virtual interrupts may also delay the timer event (if they are fired just before *VIRQ_timer*). Secondly, the CPU scheduler may delay the virtual timer interrupt until the receiver VCPU will be scheduled again. Further, Xen creates a *virtual time* which is synchronized with the hypervisor real time on every VIRQ_timer interrupt. This normally does not introduce any shift. However, in some cases the absolute timer timeout may be calculated before the virtual time synchronization but scheduled after, so that would make the timer event shift. Finally, the clock event device is optimized (similarly as in the hypervisor) with the *TIMER_SLOP* constant. All timers falling in the range of a single *TIMER_SLOP* are handled by the same single interrupt. The constant is set to 100*μs* which is fine in many cases but in some applications may be too large. For example, we have implemented a network scheduler which required timer events with resolution of tens of microseconds. In this case proposed value would be 10*μs*.

## New High Resolution Timer

To overcome the precision problems we decided to create our own timer implementation, which might be used only in cases when it is really required. For example in applications highlighted in the introduction other system processes might still use standard timer implementation built in the virtualization platform.

The idea was to introduce a new virtual interrupt *VIRQ_custom_timer* which would be handled before any other interrupts. Moreover, the other interrupts, as well as VCPU rescheduling, should be avoided near the custom timer timeout, so that it would not be disturbed. To achieve this we created a separate event channel to allow any guest domain to bind and register the *VIRQ_custom_timer* handler. This was done by defining the new virtual interrupt and increasing the number of all VIRQs in the Xen hypervisor header file xen/include/public/xen.h.

The new virtual interrupt should be delivered to a single, predefined VCPU, rather than to any of them, so that the guest domain can expect the interrupt and avoid all other interrupts near the timer deadline only on this predefined VCPU. This is possible when the function *virq_is_global* returns 0 for the *VIRQ_CUSTOM_TIMER* parameter (Listing A in S1 Text).

Such binding of the VIRQ to a single chosen virtual processor can also help to avoid the VCPU reschedules that can be achieved in two different ways depending on the application being run and the amount of available resources:

- By assigning a single physical processor to the single VCPU which is responsible for handling timer interrupts, thus disabling the VCPU scheduler completely on this virtual processor (the *vcpu-pin* command).
- By modifying the Xen CPU scheduler to ensure that the correct VCPU is scheduled near the timer deadline.

The first method requires much less modifications and does not depend on the CPU scheduler algorithm, therefore it can be recommended (especially for the multicore systems). This method was also used for the purpose of this study.

The final hypervisor modification is the actual timer scheduling and handling functionality. The guest operating systems are allowed to schedule the new timer events via the same *hypercall* (i.e. *VCPUOP_set_singleshot_timer*), as in the case of standard timers but with an additional flag *SS_CUSTOM_TIMER*.

The modified version of *VCPUOP_set_singleshot_timer* uses the existing Xen LAPIC timer functions defined in *xen/timer.h* to schedule timer events at the requested time. Finally, the hypervisor handler—*custom_timer_fn* sends the *VIRQ_CUSTOM_TIMER* interrupt to appropriate guest VCPU. These modifications are presented in Listing B in S1 Text.

All above modifications to the hypervisor kernel allow the guest domain to bind and handle the new timer events. Listing C in S1 Text presents an exemplary code which schedules and handles such a timer. This code can be used in any kernel module and allows for scheduling and handling the new timer events.

As it was already stated, it is important to treat the *VIRQ_CUSTOM_TIMER* interrupt with highest priority, to achieve the best precision. This can be set in the logic of initiating the virtual interrupt handler, in the virtual machine kernel, using the *xen_set_irq_priority* which is presented in Listing D in S1 Text.

Finally the most important change is to avoid execution of other interrupts near the timer deadline. This can be done in the *xen_evtchn_do_upcall* function as shown in Listing E in S1 Text.

Because this implementation will not be used as a general clock event device, all other timer requests will not use this functionality. This is a great advantage because the *timer_slop* variables can be left untouched and the system will not be flooded by different timer interrupts.

To verify our timer implementation and compare it with the native implementation tested previously we performed again the same set of experiments, using the new timer. The detailed results for all tested scenarios are shown in Table 1 (the “new timers” rows relate to the proposed timer implementation).

As we can note, the new timer has no problem to achieve resolutions up to 10*μs*. What is more, a significant improvement in the precision (compared to the native Xen) is gained. Especially for the system with heavy IO load the precision may be improved in some cases by two orders of magnitude (e.g., for 50*μs* we have StdDev = 0.156*μs* versus 17.628*μs* in the native Xen).

**Fig 16:**
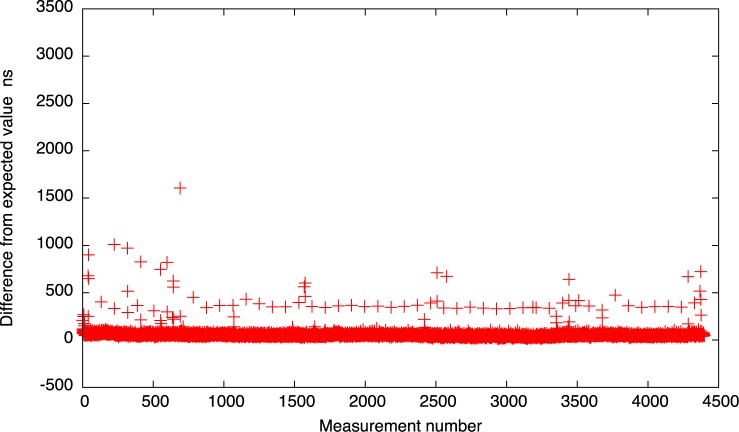
The difference between the measured timer interval and its expected value 10*μs* for the new timer implementation (idle system).

**Fig 17:**
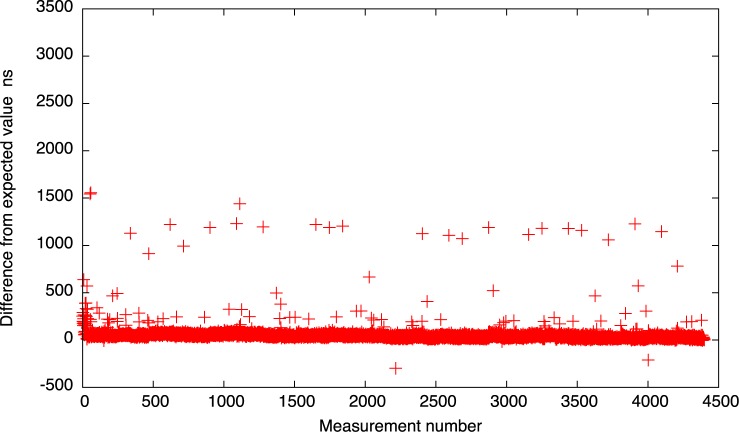
The difference between the measured timer interval and its expected value 10*μs* for the new timer implementation (system with heavy IO load).

The results for the 10*μs* setting are depicted also in Figs 16 and 17. As we can see, for both idle system and system with heavy IO operations, the results look much better than for the native Xen implementation. All the events are gathered near the expected time and only a few measurements cross the 1*μs* delay.

**Fig 18:**
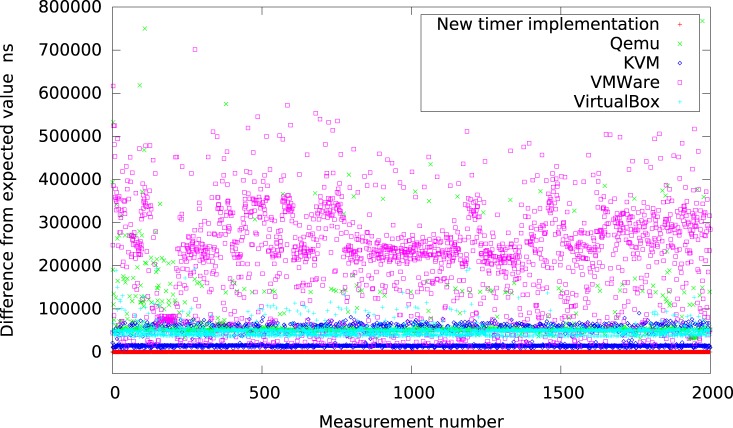
The difference between the measured timer interval and its expected value 50*μs* for platforms with low resolution (systems with heavy IO load).

**Fig 19:**
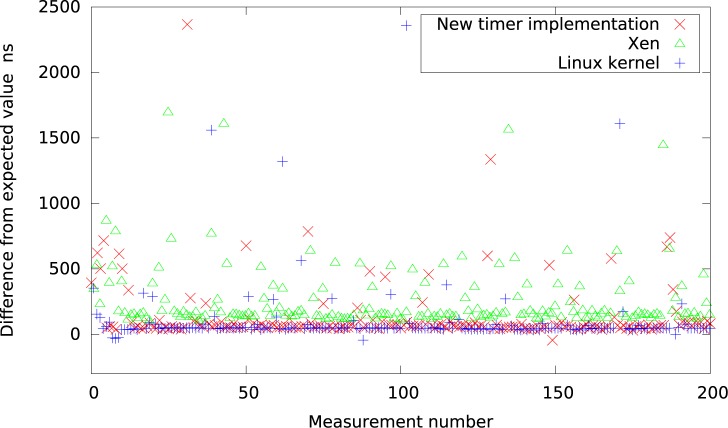
The difference between the measured timer interval and its expected value 50*μs* for platforms with higher resolution (systems with heavy IO load).

Finally, Figs 18 and 19 present all the results for intervals of 50*μs* in the system with high IO load gathered on one graph to allow visual comparison between all considered virtualization systems. In particular, in Fig 18 the new timer implementation is compared with platforms of low resolution (Qemu, KVM, VMWare and VirtualBox), while in Fig 19 with platforms of higher resolution (Xen and Linux kernel).

## Conclusion

We presented a study on high resolution timer events inside virtual machines using different virtualization platforms, i.e. Xen, KVM, Qemu, VirtualBox and VMWare. As shown in our experiments the timer resolution provided by those VMMs may not be sufficient for some purposes, especially under high interrupt load.

In particular, VMWare provided the worst resolution and precision of the timer events. KVM provided better resolution than VMWare, but still at the level of tens of microseconds. Resolution provided by Xen can be of ten microseconds but this requires changing the slop value to 1*ns* in the hypervisor and in the guest domain kernel sources. Without these changes the resolution is of a hundred microsecond. On the other hand, such changes may have a deep impact on the overall system behavior and performance.

In all studied platforms, the timer precision was strongly affected by other IO interrupts.

In order to enhance resolution and significantly reduce the impact of other IO interrupts on the precision we proposed a new timer implementation based on Xen VMM. We believe that modifications based on the same general idea can be applied analogically in the standalone Linux kernel, as well as in KVM.

Creating a separate purpose-built timer event device may prove to be the only solution for very demanding applications. In the case of open source platforms, this can be achieved by modifying the sources. However, in the case of closed solutions, such as VMWare it is not possible. As shown in the experiments, the new implementation in Xen provides a very good resolution and precision even in highly interrupted system.

## Supporting Information

S1 Text Code listings. File xen/common/event_channel.c (**Listing A**). File xen/common/domain.c (**Listing B**). Sample timer use (**Listing C**). File arch/x86/xen/time.c (**Listing D**). File drivers/xen/events/events_base.c (**Listing E**).
(PDF)
