# Supplementary material for: Achieving High Resolution Timer Events in Virtualized Environment
Source: PLoS One. 2015 Jul 15;10(7):e0130887. doi: 10.1371/journal.pone.0130887 (PMC4503740; doi:10.1371/journal.pone.0130887)
Supplement: S1 Text — File xen/common/event_channel.c (Listing A). File xen/common/domain.c (Listing B). Sample timer use (Listing C). File arch/x86/xen/time.c (Listing D). File drivers/xen/events/events_base.c (Listing E). (PDF) [file pone.0130887.s001.pdf]

Supporting Information

```
...
static int virq_is_global(uint32_t virq)
{
    int rc;

    ASSERT(virq < NR_VIRQS);

    switch ( virq )
    {
    case VIRQ_TIMER:
    //added the following line:
    case VIRQ_CUSTOM_TIMER:
    //////////////////////////
    case VIRQ_DEBUG:
    case VIRQ_XENOPROF:
        rc = 0;
        break;
    case VIRQ_ARCH_0 ... VIRQ_ARCH_7:
        rc = arch_virq_is_global(virq);
        break;
    default:
        rc = 1;
        break;
    }

    return rc;
}
...
```

Listing A. xen/common/event_channel.c

```
...
//Code added: /////////////////////////////////
struct timer custom_timer;
short initialized=0;

static void custom_timer_fn(void *data)
{
    struct vcpu *v = data;
    send_guest_vcpu_virq(v, VIRQ_CUSTOM_TIMER);
}
//////////////////////////////////////////////

long do_vcpu_op(int cmd, int vcpuid, XEN_GUEST_HANDLE(void) arg)
{
        ...
        case VCPUOP_set_singleshot_timer: {
            struct vcpu_set_singleshot_timer set;

            if (v != current)
                return -EINVAL;

            if (copy_from_guest(&set, arg, 1))
                return -EFAULT;

            //Code modified: ///////////////////////////////////////////////////
            if (set.flags == SS_CUSTOM_TIMER) {
                if (!initialized) {
                    init_timer(&custom_timer, custom_timer_fn,v, v->
                        processor);
```

```
                custom_timer.type=SS_CUSTOM_TIMER;
                initialized=1;
            }
            custom_timer.data=(void*)v;
            custom_timer.cpu=v->processor;
            stop_timer(&custom_timer);
            set_timer(&custom_timer, set.timeout_abs_ns);
        } else {
            if ( (set.flags & VCPU_SSHOTTMR_future) &&
                 (set.timeout_abs_ns < NOW()) )
                return -ETIME;
            migrate_timer(&v->singleshot_timer, smp_processor_id());
            set_timer(&v->singleshot_timer, set.timeout_abs_ns);
        }
        ////////////////////////////////////////////////////////////////////
        break;
    }
}
...
```

Listing B. xen/common/domain.c

```
...
int custom_timer_irq=-1;
unsigned long int next_timer=0;
unsigned int custom_timer_cpu=0;

static irqreturn_t custom_timer_interrupt(int irq, void *dev_id)
{
    // timer handler code
    next_timer=0;
    return IRQ_HANDLED;
}

static struct irqaction custom_timer_action = {
    .handler = custom_timer_interrupt,
    .flags   = IRQF_DISABLED|IRQF_TIMER,
    .name    = "custom_timer"
};

void schedule_custom_timer(unsigned long delay)
{
    struct vcpu_set_singleshot_timer singleshot;
    custom_timer_cpu = smp_processor_id();
    local = xen_local_clock();
    singleshot.timeout_abs_ns = local+delay;
    next_timer=local+delay;
    if (custom_timer_irq < 0) {
        custom_timer_irq = bind_virq_to_irqaction(VIRQ_CUSTOM_TIMER,
            custom_timer_cpu, &custom_timer_action);
    }
    singleshot.flags = SS_CUSTOM_TIMER;
    rc = HYPERVISOR_vcpu_op(VCPUOP_set_singleshot_timer, custom_timer_cpu,
        &singleshot);
}
...
```

Listing C. Sample timer use.

```
...
```

```
        irq = bind_virq_to_irqhandler(VIRQ_TIMER, cpu, xen_timer_interrupt
            ,
                                      IRQF_PERCPU|IRQF_NOBALANCING|
                                          IRQF_TIMER|
                                      IRQF_FORCE_RESUME|IRQF_EARLY_RESUME,
                                      name, NULL);
        //VIRQ_TIMER priority decreased by 1 below: //////////////////////
        (void)xen_set_irq_priority(irq, XEN_IRQ_PRIORITY_MAX+1);

        memcpy(evt, xen_clockevent, sizeof(*evt));

        evt->cpumask = cpumask_of(cpu);
        evt->irq = irq;
        per_cpu(xen_clock_events, cpu).name = name;

        //Code added: ////////////////////////////////////////////////
        printk(KERN_INFO "installing Xen custom timer for CPU %d\n", cpu);

        if (cpu==0) {
                init_procfs();
        }

        name = kasprintf(GFP_KERNEL, "customtimer%d", cpu);
        if (!name)
                name = "<timer kasprintf failed>";


        get_cpu_var(custom_timer_irq) = bind_virq_to_irqhandler(
            VIRQ_CUSTOM_TIMER, cpu, custom_timer_interrupt,
                                      IRQF_PERCPU|IRQF_NOBALANCING|
                                          IRQF_TIMER|
                                      IRQF_FORCE_RESUME|IRQF_EARLY_RESUME,
                                      name, NULL);
        (void)xen_set_irq_priority(get_cpu_var(custom_timer_irq),
            XEN_IRQ_PRIORITY_MAX);
        ///////////////////////////////////////////////////////////////
...
```

Listing D. arch/x86/xen/time.c

```
...
//Code added: ///////////////////////////////////////////////////
static int customtimer_evtchn=-1;
int find_customtimer_evtchn(void) {
        unsigned int evtchn;
        int irq;
        if (customtimer_evtchn==-1) {
                irq=irq_from_virq(timerCPU, VIRQ_CUSTOM_TIMER);
                for (evtchn = 0; evtchn < xen_evtchn_nr_channels(); evtchn
                    ++) {
                        if (irq==get_evtchn_to_irq(evtchn)) {
                                customtimer_evtchn=evtchn;
                                break;
                        }
                }
        }
        return customtimer_evtchn;
}
/////////////////////////////////////////////////////////////////////////

void xen_evtchn_do_upcall(struct pt_regs *regs)
{
        ...
```

```
        #ifdef CONFIG_X86
                exit_idle();
                inc_irq_stat(irq_hv_callback_count);
        #endif
        //Code added: ////////////////////////////////////////////////
        if ((smp_processor_id()==custom_timer_cpu) && next_timer) {
                unsigned int port=find_customtimer_evtchn();
                if ((xen_local_clock()>(PItimer-customtimer_before)) && (
                    port!=-1) && !test_evtchn(port)) {
                        irq_exit();
                        set_irq_regs(old_regs);
                        return;
                }
        }
        ////////////////////////////////////////////////////////////////
        __xen_evtchn_do_upcall();
        ...
}
...
```

Listing E. drivers/xen/events/events_base.c
